# Effectiveness of community-based PrEP delivery for men who have sex with men: a global systematic review and meta-analysis

**DOI:** 10.3389/fpubh.2026.1868507

**Published:** 2026-09-10

**Authors:** Abeer Teeti, Issam AbuQeis, Xuan Zhang, Xiaowen Wang

**Affiliations:** 1 Department of Epidemiology, School of Public Health, Kunming Medical University, Kunming, China; 2 School of Basic Medicine, Kunming Medical University, Kunming, China; 3 Yunnan International Joint Laboratory of Public Health and Disease Control, Yunnan Center for Disease Control and Prevention (Yunnan Academy of Preventive Medicine), Kunming, Yunnan, China

**Keywords:** community-based, HIV, meta-analysis, MSM, pre-exposure prophylaxis (PrEP)

## Abstract

Pre-exposure prophylaxis (PrEP) is a critical HIV prevention tool for men who have sex with men (MSM), used throughout as an inclusive umbrella term encompassing gay, bisexual, and other men who have sex with men (GBMSM). Yet, the global effectiveness of community-based delivery models across the prevention cascade remains unclear. This systematic review and meta-analysis (Prospero registered: CRD420261382434) provides a comprehensive global synthesis of community-based PrEP delivery for MSM. Following PRISMA guidelines, six databases were searched (2015–2023), and random-effects models were used to pool Odds Ratios from 21 studies. Community-based models significantly increased PrEP willingness (OR = 5.12, 95% CI: 4.30–6.10) and positive risk behavior changes (OR = 1.61, 95% CI: 1.33–1.96); however, pooled effects for uptake and adherence were non-significant with extreme heterogeneity (*i*^2^ > 88%). Critically, subgroup analyses revealed that effectiveness is not monolithic but context-dependent: telehealth models were exceptionally effective for uptake (OR = 25.20), and outcomes varied significantly by region and study design. Policy should support differentiated, hybrid models that combine community-based engagement with streamlined telehealth or pharmacy-led pathways, tailored to the local context.

**Systematic review registration:**

https://www.crd.york.ac.uk/PROSPERO/view/CRD420261382434, Identifier CRD420261382434.

## Introduction

HIV remains a major global public health challenge, disproportionately affecting key populations including men who have sex with men (MSM), people who inject drugs, transgender women, sex workers, serodifferent couples, and adolescent girls and young women in high-burden settings (1). Among these groups, MSM continue to experience markedly higher rates of new infection compared to the general population, particularly in regions with limited healthcare access and entrenched stigma (2). Pre-exposure prophylaxis (PrEP) is a highly effective biomedical prevention tool that, when taken with optimal adherence, reduces HIV acquisition risk by over 99% in clinical trials (3) and demonstrates comparable effectiveness in real-world community-based settings (4–6). As part of a combination prevention approach that includes condom use, harm reduction, Post-exposure Prophylaxis (PEP), and treatment as prevention (U=U), PrEP offers a user-initiated option for individuals at ongoing risk of HIV exposure (7). Yet, the practical application of PrEP encounters major obstacles, such as stigma, expense, and inefficiencies in healthcare systems, limiting its broad uptake among MSM (8). Community-based PrEP delivery models have gained traction as a promising strategy to overcome these barriers by decentralizing services and engaging priority populations in familiar, non-stigmatizing environments (9). Beyond MSM, these models are increasingly being adapted for people who inject drugs, transgender women, and adolescent girls and young women, with emerging evidence suggesting they improve engagement across diverse cultural contexts. For MSM specifically, community-driven approaches, frequently leveraging peer educators, mobile health units, or local health personnel, have shown particular promise in increasing service availability and acceptability, especially where conventional medical structures lack the capacity to support stigmatized groups (10).

Research in sub-Saharan Africa and Southeast Asia indicates that community-driven methods improve PrEP adoption and continued engagement among MSM, especially where conventional medical structures lack the capacity to support vulnerable groups (11). However, worldwide data on the efficacy of these models remain inconsistent, with notable differences in program structure, execution, and results across geographic areas (12). A critical research gap exists in synthesizing the comparative effectiveness of community-based PrEP delivery for MSM across diverse socio-cultural and healthcare contexts. While several systematic and scoping reviews have explored PrEP implementation, they are limited by significant constraints. Existing reviews have focused narrowly on specific geographical regions, such as the United States (13) or on particular intervention types, such as digital health tools (14). Others have been qualitative scoping reviews that map the landscape of service delivery models without providing a quantitative synthesis of their effectiveness (15). Crucially, no prior meta-analysis has quantitatively synthesized the global evidence for community-based PrEP delivery models for MSM across the entire prevention cascade, from willingness and uptake to adherence and risk behavior. Moreover, current reviews frequently concentrate narrowly on clinical results but fail to address the wider consequences of community engagement, cost-effectiveness, and long-term sustainability (16). Addressing these gaps is essential to inform policy and programmatic decisions, particularly in resource-limited settings where scalable and sustainable PrEP delivery solutions are urgently needed. This review focuses on community-based delivery of oral pre-exposure prophylaxis, primarily tenofovir disoproxil fumarate/emtricitabine (TDF/FTC), which represents the predominant formulation in the included studies. Newer formulations, including tenofovir alafenamide/emtricitabine (TAF/FTC) and long-acting injectable cabotegravir (LA-CAB), have emerged more recently and may introduce additional heterogeneity in delivery outcomes (17, 18).

To structure this review, we adopt the PrEP care cascade as our conceptual framework (19). This cascade maps the sequential steps required for PrEP to achieve population-level impact: awareness and willingness to use PrEP; uptake and initiation; adherence and retention in care; and ultimately, sustained risk reduction and prevention outcomes. Analyzing community-based delivery models through this cascade allows us to identify precisely where in the continuum these models exert their strongest effects and where implementation bottlenecks persist. While prior reviews have examined isolated segments of this cascade, no meta-analysis has quantitatively synthesized evidence across all stages for MSM globally. This review advances academic discourse by conducting the inaugural comprehensive systematic review and meta-analysis of worldwide data on community-based PrEP distribution among MSM. We synthesize data from diverse geographical and socio-economic settings, including Asia, Africa, Europe, North America, and South America, to assess the impact of these models on five key outcome domains: PrEP coverage and adherence, uptake, willingness, program-related outcomes, and changes in sexual risk behavior. Our results hold important consequences for decision-makers, program administrators, and activists aiming to improve PrEP distribution for MSM globally. Through the recognition of primary enablers and obstacles, this review progresses the comprehension of how approaches rooted in communities can be adapted to optimize their effect on public health.

## Methodology

### Review protocol and search strategy

This systematic review and meta-analysis adhered to the PRISMA guidelines (20) to uphold methodological precision and clarity. We conducted comprehensive searches across five major databases and one search engine, prioritized based on their relevance to biomedical and public health research. PubMed was selected as the primary database due to its extensive coverage of medical literature and advanced MeSH term functionality. Web of Science was included for its multidisciplinary scope and citation tracking capabilities. Scopus delivered extra scope with its extensive collection of abstracts and citations. ScienceDirect was chosen for its strong collection of full-text articles in health sciences. Embase supplemented these with its repository of peer-reviewed journals. Google Scholar served as an additional search method to locate grey literature and guarantee the inclusion of all pivotal studies.

The search strategy employed a combination of controlled vocabulary (e.g., MeSH terms) and free-text keywords to maximize sensitivity and specificity. The PubMed search strategy merged (“Community-Based” [MeSH] AND “Pre-Exposure Prophylaxis” [MeSH]) OR (“Community-Based” [MeSH] AND PrEP [TIAB]) with (“Men Who Have Sex with Men” [MeSH] OR MSM [TIAB]), while omitting review articles and surveys. Similar logic was applied across other databases with platform-specific syntax adjustments. The search was limited to studies published between January 2015 and October 2025 to capture contemporary evidence on PrEP implementation. The search was limited to studies published between January 2015 and October 2025. The year 2015 was selected as the starting point because it corresponds with the World Health Organization’s 2015 guidelines recommending PrEP for MSM (21), marking the transition from clinical trials to widespread community-based implementation. While oral TDF/FTC received FDA approval in 2012, the period 2012–2014 primarily comprised clinical trial and early demonstration project data, whereas our review specifically targets community-based delivery models in implementation settings. Supplementary Table S1 contains the complete search syntax details for every database.

For search strategy validation and terminology variations, the search syntax was optimized for PubMed’s MeSH and free-text term functionality and may yield varying result counts depending on the search date and platform. We acknowledge that alternative terminology, including “gay, bisexual, and other men who have sex with men (GBMSM)”, may retrieve additional studies not captured by “MSM” alone. To mitigate this, we employed comprehensive free-text and MeSH term combinations and manually screened reference lists of included studies and relevant reviews to identify additional eligible publications. The potential impact of terminology variation on study retrieval is acknowledged as a limitation.

### Inclusion and exclusion criteria

Inclusion criteria were pre-specified and applied systematically to ensure reproducibility:

- *Population*: Men who have sex with men (MSM) aged ≥18 years, including studies with mixed populations where MSM data could be extracted separately.
- *Intervention*: Community-based PrEP delivery models, defined as programs conducted outside conventional clinical settings, including peer-led, mobile health, telehealth-integrated, pharmacy-based, and community organization-led models.
- *Comparator*: Standard clinical care or any alternative PrEP delivery model.
- *Outcomes*: Quantitative data on PrEP willingness, uptake, adherence, coverage, program-related outcomes, or sexual risk behavior changes.
- *Study design*: Original quantitative or mixed-methods research (with extractable quantitative data); randomized controlled trials, cohort studies, and cross-sectional studies.
- *Language*: English-language publications only.
- *Timeframe*: Published between January 2015 and October 2025.

### Exclusion criteria

- Studies conducted exclusively in conventional clinical settings without community-based components.
- Studies focusing exclusively on non-MSM populations (unless subgroup data for MSM were available).
- Qualitative-only studies, case reports, editorials, commentaries, and conference abstracts.
- Studies without primary outcome data or those reporting only qualitative findings.
- Review articles and systematic reviews (used only for reference list screening).

### Study selection and eligibility criteria

**Figure 1:**
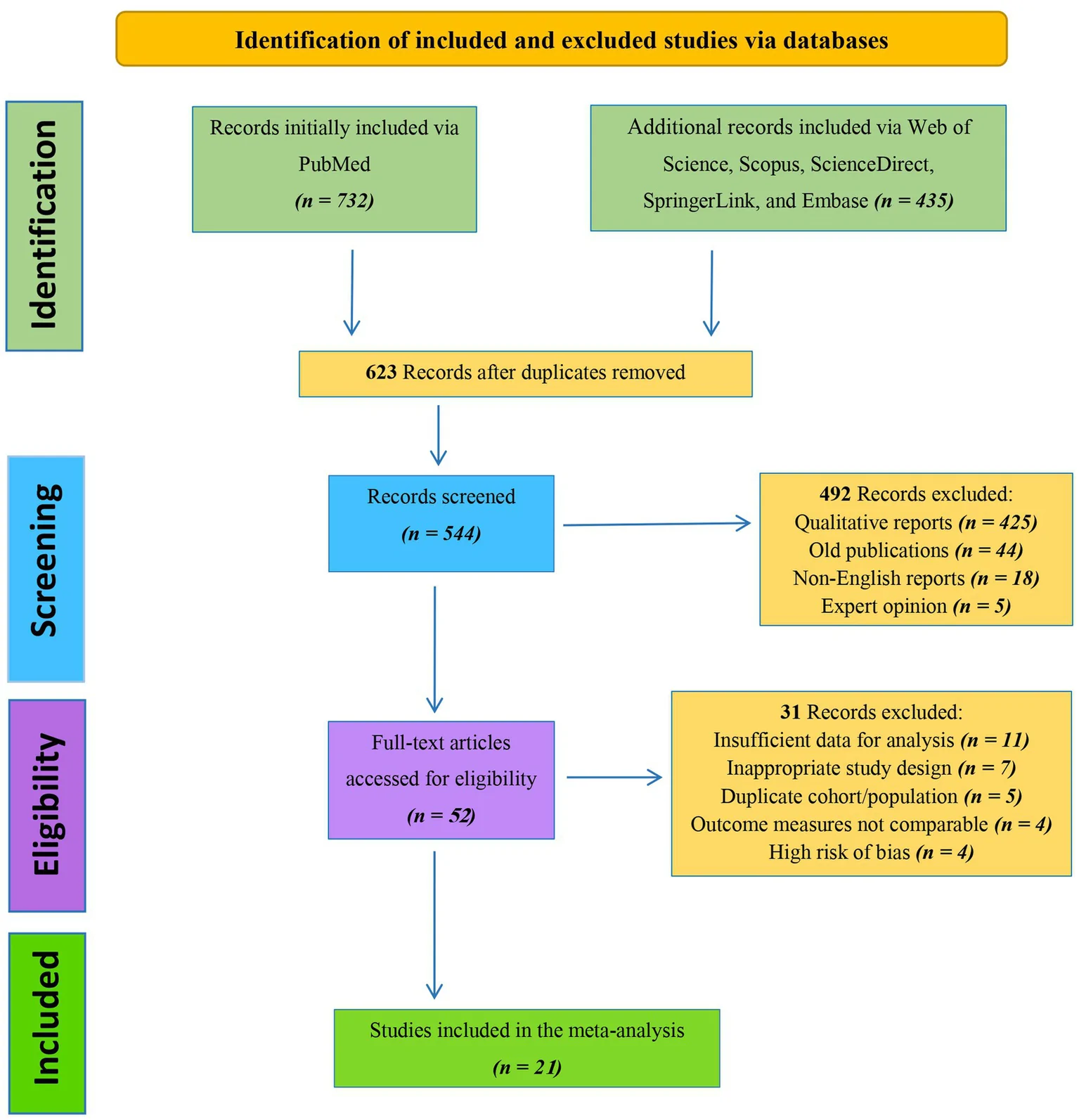
PRISMA flowchart of study selection process. Identification of included and excluded studies via databases, detailing the flow of study records through identification, screening, eligibility assessment, and inclusion stages, with corresponding counts.

The selection procedure consisted of three consecutive screening stages, each carried out separately by two assessors (WX and IA) with discrepancies resolved by a third reviewer (AT). Initial deduplication removed 623 records from the 1,167 identified through database searches. Screening the titles and abstracts of the remaining 544 records resulted in the exclusion of 492 publications that failed to meet the basic eligibility criteria. A full-text review of 52 potentially relevant articles was conducted, and 31 were excluded because they did not meet all inclusion requirements. Review quality was assessed with the PRISMA checklist (Supplementary File S2) and the Joanna Briggs Institute (JBI) critical appraisal checklist (22) for systematic reviews and research synthesis (Supplementary File S3), focusing on methodological risks of bias (ROB), which were assessed using tools specific to the study design (Supplementary File S4). For randomized controlled trials, we used the Cochrane Risk of Bias 2 (RoB 2) tool (23). For observational studies (cohort and cross-sectional), we employed the NIH Quality Assessment Tool (24), evaluating key domains such as selection bias, confounding, measurement validity, and attrition. Studies were graded as ‘Low Risk’, ‘Moderate Risk’ (Some Concerns), or ‘High Risk’ based on critical flaws (e.g., attrition rates >20% or unmitigated selection bias in online surveys). The concluding analysis comprised 21 research papers fulfilling all quality criteria (Figure 1).

The selection process may have drawbacks, such as language bias due to the exclusion of non-English publications and the potential lack of studies from low-resource settings. Observational designs (cross-sectional and cohort) being most common in the final sample may introduce confounding that could not be entirely resolved by statistical adjustment. Publication bias was formally assessed and reported to quantify potential overrepresentation of positive findings.

## Results

### Overview of included studies

The systematic review identified 21 studies that satisfied the inclusion criteria, which covered various geographical areas and community-driven PrEP distribution approaches for MSM. Six outcomes were measured via the Odds Ratio and assessed via effect size results. These metrics were selected to capture both clinical and implementation outcomes relevant to community-based PrEP programs. Supplementary Table S5 presents the coded outcomes from the included studies, categorized by study design, sample size, geographical location, and effect size measures. The research included cross-sectional (*n* = 11), cohort (*n* = 8), and randomized controlled trial (*n* = 2) designs, with sample sizes ranging from 18 to 3,924 participants. The studies covered regions including Asia (*n* = 8), North America (*n* = 7), Africa (*n* = 3), Europe (*n* = 3), and South America (*n* = 1), which illustrates the worldwide reach of community-driven PrEP initiatives.

### Outcome definitions and domain consolidation

Outcomes were categorized into five domains aligned with the PrEP care cascade: (1) PrEP willingness (self-reported intent or interest in using PrEP); (2) PrEP uptake (initiation of PrEP among eligible or willing individuals); (3) PrEP coverage (proportion of the eligible population accessing PrEP services); (4) PrEP adherence (medication-taking behavior measured via self-report, pharmacy refill, or drug-level testing); (5) program-related outcomes (retention in care, persistence, and clinic attendance); and (6) changes in sexual risk behavior (changes in condom use, number of partners, or other risk indicators).

We acknowledge that PrEP coverage and adherence represent conceptually distinct constructs: coverage reflects the reach of services at the population level (i.e., the proportion of eligible individuals who access PrEP), whereas adherence reflects individual-level medication-taking behavior once PrEP has been initiated. However, in the context of this review, these outcomes were consolidated into a single meta-analytic domain for two methodological reasons. First, the included studies did not uniformly distinguish between these constructs; several studies reported composite metrics (e.g., “proportion of enrolled participants with detectable drug levels”) that simultaneously captured both access to PrEP and ongoing use, precluding clean separation. Second, both coverage and adherence represent proximal implementation outcomes along the PrEP care continuum, positioned after uptake and before sustained behavioral change, and are frequently analyzed together in implementation science frameworks as indicators of program engagement and maintenance. This consolidation is consistent with prior meta-analytic approaches in HIV prevention implementation science, in which related proximal outcomes have been pooled when individual-study reporting precluded discrete separation.

### Data analysis and synthesis

The main analysis revealed substantial to extreme heterogeneity across all outcome domains, with *i*^2^ values ranging from 69 to 99% (Table 1). This high level of inconsistency indicated that a single pooled effect size was insufficient to represent the diverse results across studies. Consequently, to explore the sources of this variation, we conducted pre-specified subgroup analyses based on key review-level characteristics hypothesized to influence the intervention’s effectiveness. Where odds ratios were not directly reported in the original study, they were calculated from available 2 × 2 contingency table data using the formula OR = (a × d)/(b × c), with calculations verified by two independent reviewers. Studies without a comparator group or sufficient raw data were excluded from quantitative synthesis and reported narratively. These subgroup comparisons were selected based on established theoretical and empirical precedents. First, the country income level was chosen because healthcare infrastructure, supply chain reliability, and community health worker capacity differ systematically between high-income and low/middle-income settings, directly affecting adherence support. Second, the delivery model (peer-led, telehealth, hybrid) was selected as a proxy for the underlying mechanism of action: telehealth reduces structural barriers such as travel and clinic stigma, while peer-led models operate through social network diffusion and trust, suggesting differential effects on uptake. Third, study design was included because cross-sectional surveys of willingness are susceptible to social desirability and selection bias, whereas cohort designs track temporal changes, potentially yielding more conservative effect estimates. Finally, for PrEP-related outcomes, we pre-specified a sensitivity analysis to assess the influence of any single dominant study, as large programmatic evaluations can disproportionately drive pooled estimates and mask effects observed in smaller, targeted interventions.

**Table 1:** Heterogeneity statistics for main and subgroup outcome measures.

Subgroups	*Q*	*i*^2^ (%)	*p*-value	*τ* ^2^
PrEP coverage and adherence	58.39	90	<0.00001	1.16
Low and middle-income	38.01	92	<0.00001	–
High income	3.34	40	= 0.19	–
PrEP uptake	255.59	98	<0.00001	3.78
peer_led	13.25	92	= 0.0003	–
Telehealth	38.26	97	<0.00001	–
Hybrid	N/A	N/A	N/A	–
PrEP willingness	280.98	99	<0.00001	3.22
Cohort	2.01	50	= 0.16	–
Cross sectional	265.38	99	<0.00001	–
PrEP-related outcomes	39.95	92%	0.00001	2.73
Sensitivity test	1.17	0	= 0.56	–
Changes in risk behaviors	6.43	69%	0.04	0.02

*Q* = Cochran’s *Q* statistic (Chi^2^); *i*^2^ = inconsistency index; *τ*^2^ = between-study variance; N/A = not applicable (single study in subgroup).

For PrEP coverage and adherence, we stratified studies by income level (Low and middle vs. High). For PrEP uptake, we categorized interventions by delivery model (peer-led, telehealth, or hybrid). For PrEP willingness, we grouped studies by their design (cohort vs. cross-sectional). Furthermore, a preliminary analysis of PrEP-related outcomes revealed that one study (25) contributed disproportionately to the pooled estimate (89.5% of the total weight), potentially masking the effects observed in other studies. Therefore, we conducted a sensitivity analysis for this outcome by excluding this single influential study to assess its impact on the overall effect size and heterogeneity. All analyses were conducted using random-effects models to account for anticipated between-study variance.

### Meta-analysis

The meta-analysis synthesized evidence across five key outcome domains to evaluate the effectiveness of community-based PrEP delivery for MSM. Random-effects models were applied to address the considerable variability noted among studies, and effect sizes were aggregated employing appropriate metrics for each outcome. The examination provides a comprehensive assessment of the impact of community-driven methods on PrEP coverage, adherence, uptake, willingness, related outcomes, and change in risk behavior across diverse international contexts.

### Main outcomes

#### PrEP willingness

**Figure 2:**
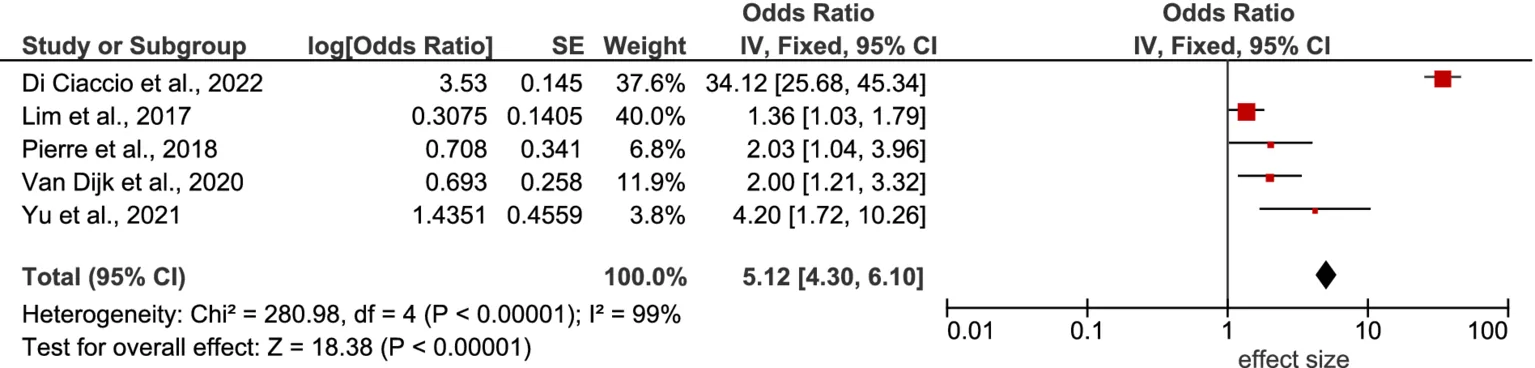
Forest plot for PrEP willingness. This plot illustrates the effect of community-based PrEP delivery on willingness to use PrEP across five studies. The pooled odds ratio was statistically significant (*OR* = 5.12, 95% *CI*: 4.30–6.10, *p* < 0.00001), with extreme heterogeneity observed (*i^2^* = 99%, *p* < 0.00001).

The meta-analysis of PrEP willingness across five studies demonstrated a statistically significant and positive pooled effect. The OR was 5.12 (95% CI, 4.30–6.10, *p* < 0.00001), indicating that, on average, individuals exposed to community-based PrEP delivery models had over five times higher odds of expressing willingness to use PrEP compared to those in standard care settings. The forest plot (Figure 2, Supplementary Table S6) shows extreme heterogeneity among the studies (*i*^2^ = 99%, χ^2^ = 280.98, *p* < 0.00001), suggesting that while the direction of effect is consistently positive, the magnitude of impact varies substantially across implementations. However, all five individual studies reported point estimates favoring community-based models, with confidence intervals that did not cross the line of no effect (OR = 1). The effects ranged from a modest increase, as seen in Lim et al. (26) (OR = 1.36, 95% CI: 1.03–1.79), to more substantial increases in studies like Pierre et al. (27) (OR = 2.03) and Van Dijk et al. (28) (OR = 2.00). The strongest effect was reported by Di Ciaccio et al. (29), who found an exceptionally high association (OR = 34.12, 95% CI: 25.68–45.34), followed by Yu et al. (30) (OR = 4.20, 95% CI: 1.72–10.26). The extreme heterogeneity likely reflects vast differences in target populations, the specific messaging or education components of the programs, or cultural contexts. Still, it does not detract from the clear overall beneficial effect.

#### PrEP uptake

**Figure 3:**
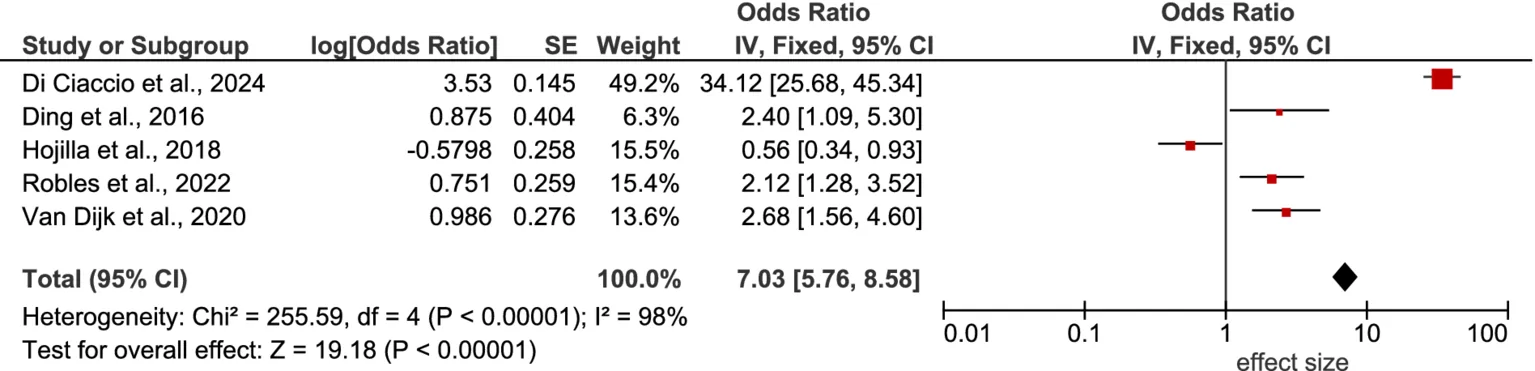
Forest plot for PrEP uptake. This plot illustrates the effect of community-based PrEP delivery on uptake across five studies. The pooled odds ratio was not statistically significant (*OR* = 3.06, 95% *CI*: 0.55–17.13, *p* = 0.20), with extreme heterogeneity observed (*i^2^* = 98%, *p* < 0.00001).

The meta-analysis of PrEP uptake across five studies yielded a pooled OR of 3.06 (95% CI, 0.55–17.13, *p* = 0.20), which was not statistically significant. The forest plot (Figure 3, Supplementary Table S7) reveals extreme heterogeneity in effect sizes (*i*^2^ = 98%, *τ*^2^ = 3.78, χ^2^ = 255.59, *p* < 0.00001), signifying that the impact of community-based PrEP programs varied extraordinarily across different implementations. This variation is starkly evident in the opposing results of individual studies. One study, Hojilla et al. (31), reported an effect significantly favoring standard care, with community-based models associated with reduced odds of uptake (OR = 0.56, 95% CI: 0.34–0.93). In contrast, the remaining four studies demonstrated positive effects, though of vastly different magnitudes. Robles et al. (32), Van Dijk et al. (28), and Ding et al. (33) (Those who had male sex partners in their lifetime were significantly more likely to participate in the tenofovirdisoproxil fumarate group) found moderate benefits, with ORs ranging from 2.12 to 2.68. Most strikingly, Di Ciaccio et al. (29) reported an exceptionally strong positive effect (OR = 34.12, 95% CI: 25.68–45.34).

#### PrEP coverage and adherence

**Figure 4:**
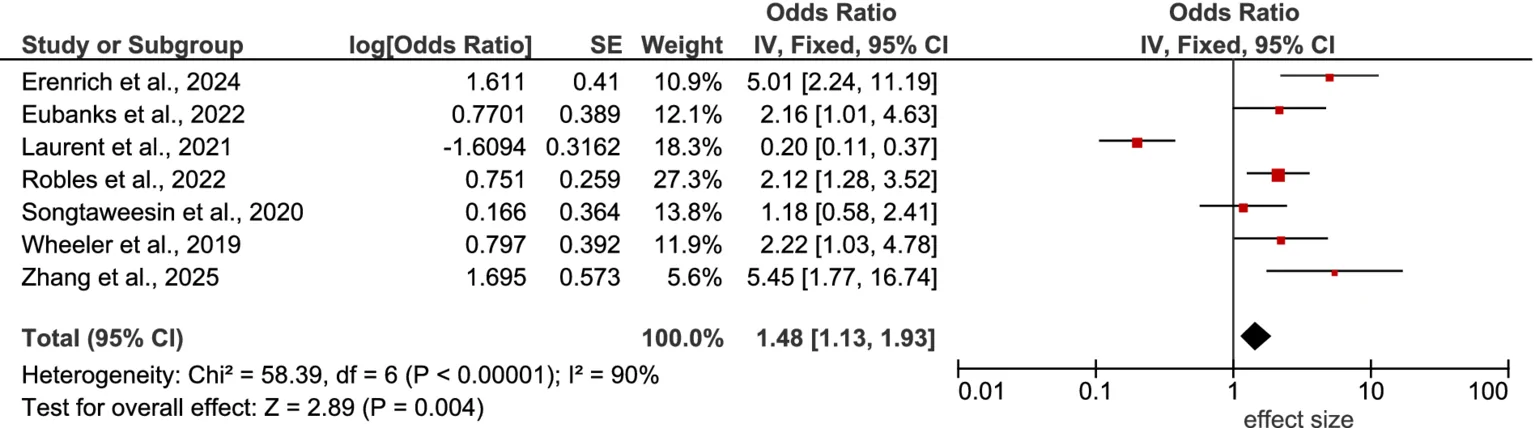
Forest plot for PrEP coverage and adherence. This plot illustrates the impact of community-based PrEP delivery on coverage and adherence outcomes across seven studies. The pooled odds ratio was not statistically significant (OR = 1.48, 95% CI: 1.13–1.93, *p* = 0.004), with considerable heterogeneity observed across the included studies (*i*^2^ = 90%, *p* < 0.00001).

The meta-analysis of PrEP coverage and adherence across seven studies yielded a pooled Odds Ratio (OR) of 1.48 (95% CI, 1.13–1.93, *p* = 0.004), which was not statistically significant. The forest plot (Figure 4, Supplementary Table S8) reveals substantial heterogeneity in effect sizes (*i*^2^ = 90%, *τ*^2^ = 1.16 (calculated), χ^2^ = 58.39, *p* < 0.00001), indicating that the impact of community-based models varied dramatically across different settings and populations. This variation is highly evident in the study-specific results. For instance, a study reported effects significantly favoring standard care; Laurent et al. (34) Similarly, one study reported significantly reduced odds (OR = 0.20, 95% CI: 0.11–0.37). In contrast, three studies demonstrated statistically significant positive effects. Positive outcome, as mirrored by Wheeler et al. (35), who also found a statistically significant association favoring the intervention. The strongest evidence was reported by Ehrenrich et al. (36) and Zhang et al. (37), who found even larger effect sizes (OR = 5.01, 95% CI: 2.24–11.19 and OR = 5.45, 95% CI: 1.77–16.74, respectively). Three studies yielded inconclusive results. Songtaweesin et al. (38) reported an effect size near unity, indicating no significant difference between the delivery models. Meanwhile, Eubanks et al. (39) and Robles et al. (32) displayed positive trends favoring community-based delivery, but their results did not reach statistical significance because the confidence intervals crossed the no-effect line.

#### PrEP-related outcomes

**Figure 5:**
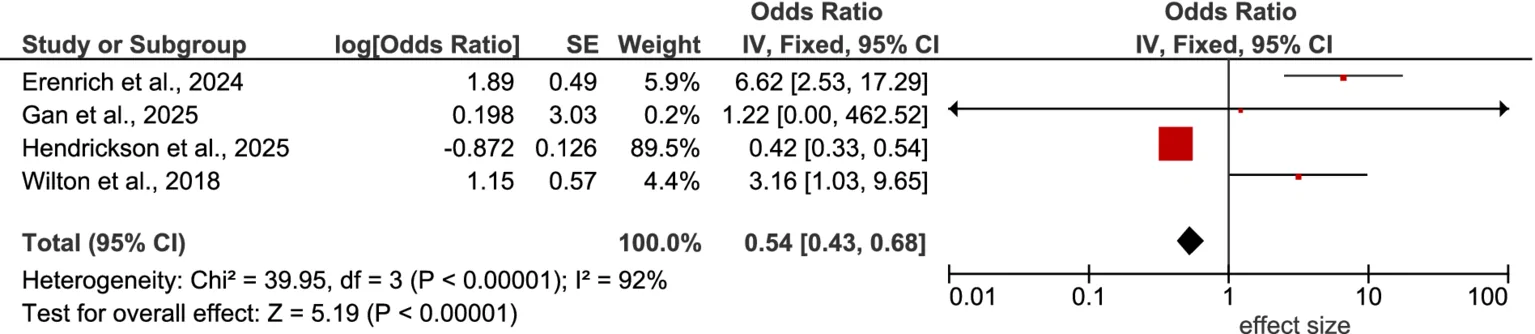
Forest plot for PrEP-related outcomes. This plot shows the effect of community-based PrEP delivery on intermediate program outcomes across four studies. The pooled odds ratio was statistically significant, favoring standard care (OR = 0.54, 95% CI: 0.43–0.68, *p* < 0.00001), with high statistical heterogeneity observed (*i*^2^ = 92%, *p* < 0.00001).

The meta-analysis of PrEP-related outcomes across four studies demonstrated a statistically significant pooled effect favoring standard clinical care over community-based models. The pooled OR was 0.54 (95% CI, 0.43–0.68, *p* < 0.00001). This indicates that, in this specific grouping of studies, community-based delivery was associated with 46% lower odds of achieving the evaluated positive outcomes compared to standard care. The forest plot (Figure 5, Supplementary Table S9) reveals high statistical heterogeneity among the included studies (*i*^2^ = 92%, χ^2^ = 39.95, *p* < 0.00001). Meanwhile, this overall result is heavily influenced by the study by Hendrickson et al. (25), which contributed 89.5% of the total weight and reported significantly reduced odds for the intervention group (OR = 0.42, 95% CI: 0.33–0.54). In contrast, the other three studies favored community-based models: Erenrich et al. (36) (OR = 6.62) and Wilton et al. (40) (OR = 3.16) showed significant positive effects, while Gan et al. (41) showed a point estimate favoring the intervention (OR = 1.22) but with a very wide confidence interval.

#### Change in risk behavior

**Figure 6:**
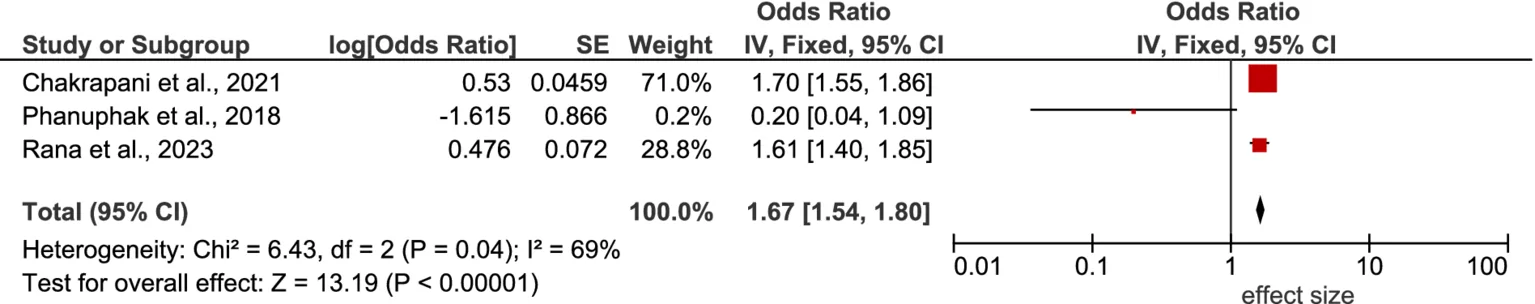
Forest plot for change in risk behavior. This plot illustrates the effect of community-based PrEP delivery on sexual risk behavior across three studies. The pooled odds ratio was statistically significant (OR = 1.61, 95% CI: 1.33–1.96, *p* < 0.00001), with substantial heterogeneity observed (*i*^2^ = 69%, *p* = 0.04).

The meta-analysis of changes in sexual risk behavior across three studies demonstrated a statistically significant pooled effect favoring community-based models. The OR was 1.61 (95% CI, 1.33–1.96, *p* < 0.00001), indicating that, on average, participation in a community-based PrEP program was associated with 61% higher odds of reporting decreased or stable risk behavior compared to standard care. The forest plot (Figure 6, Supplementary Table S10) reveals substantial heterogeneity in effect sizes (*i*^2^ = 69%, χ^2^ = 6.43, *p* = 0.04), reflecting inconsistency in the magnitude of observed behavioral changes across different study contexts. Two studies, which together comprise 98.7% of the total analysis weight, reported significant effects favoring reduced risk practices; Chakrapani et al. (42) found a 70% increase in the odds of positive behavioral outcomes (OR = 1.70, 95% CI: 1.55–1.86). Rana et al. (43), reported a similar 61% increase (OR = 1.61, 95% CI: 1.40–1.85). In contrast, Phanuphak et al. (44) reported a point estimate suggesting a potential increase in risk behavior (OR = 0.20, 95% CI: 0.04–1.09). However, its confidence interval is wide and crosses the line of no effect (OR = 1.0), rendering the result statistically non-significant. This review contributed minimal weight (1.3%) to the pooled result due to its lower precision. The observed heterogeneity underscores that behavioral responses are not uniform and may be influenced by specific program components or cultural norms.

#### Subgroup and sensitivity analyses

**Figure 7:**
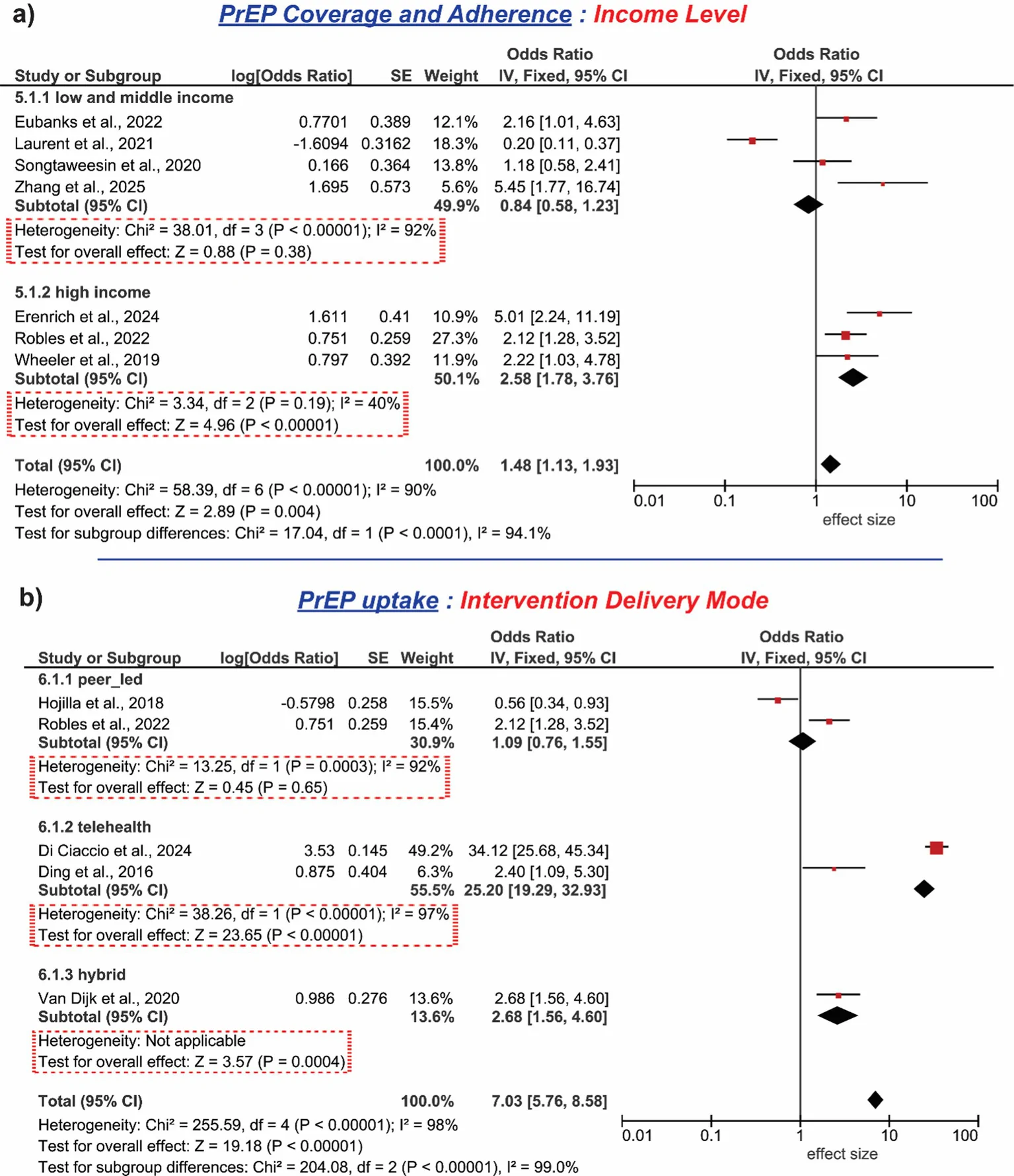
Forest plots of the subgroup for PrEP outcomes. The pooled odds ratios (OR) are shown for: **(A)** PrEP coverage and adherence, stratified by country income level (low/middle income vs. high income); **(B)** PrEP uptake, stratified by intervention delivery mode (peer-led, telehealth, hybrid).

**Figure 8:**
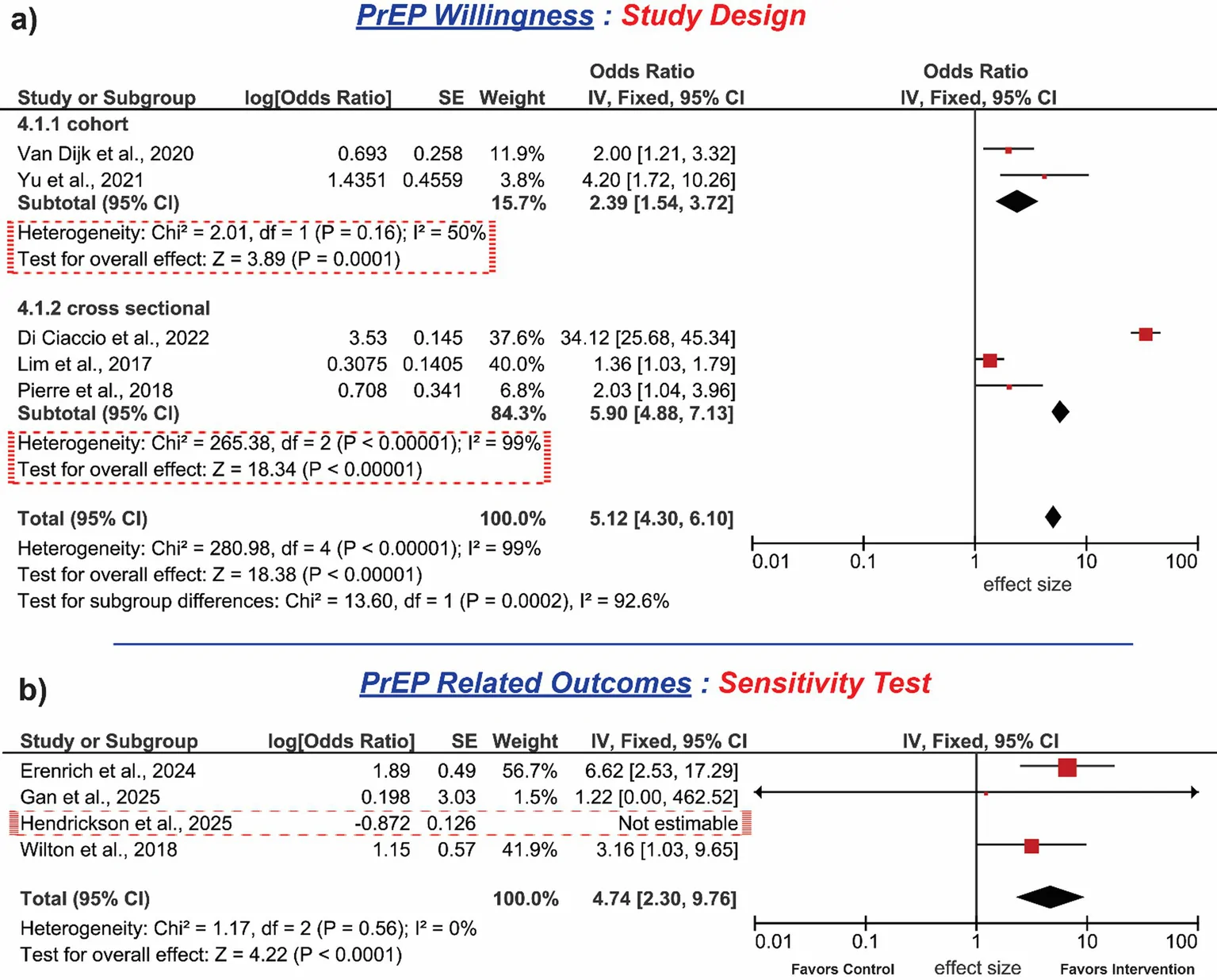
Forest plots of the subgroup and sensitivity analyses for PrEP outcomes. **(A)** PrEP willingness, stratified by study design (cohort vs. cross-sectional). **(B)** Sensitivity analysis for PrEP-related outcomes before and after exclusion of an influential outlier study (25). Diamonds represent pooled ORs for each subgroup; width indicates 95% confidence intervals. All analyses used a fixed-effects model. Heterogeneity statistics and subgroup difference tests are shown.

To investigate the profound heterogeneity observed in the main analyses, we conducted several subgroup and sensitivity analyses (Figure 7 and Figure 8, Supplementary Table S11). For PrEP coverage and adherence, subgroup analysis by income level revealed a significant difference (*p* = 0.004). Studies conducted in high-income settings showed a strong, significant positive effect (OR = 2.58, 95% CI: 1.78–3.76), whereas studies in low- and middle-income countries showed a much smaller, albeit still significant, benefit (OR = 0.84, 95% CI: 0.58–1.23) (Figure 7A). This suggests that the effectiveness of community-based models for adherence and coverage is significantly greater in high-income contexts, which partially explains the moderate heterogeneity (*i*^2^ = 50.1%) observed in the overall analysis. The subgroup difference test confirmed substantial heterogeneity between income levels (Chi^2^ = 3.34, df = 2, *p* < 0.0001; *i*^2^ = 90%). For PrEP uptake, the analysis revealed a highly significant difference between intervention types (*p* < 0.00001). Telehealth models were exceptionally effective (OR = 25.20, 95% CI: 19.29–32.93), demonstrating a powerful impact on uptake. Hybrid models also showed a significant benefit (OR = 2.68, 95% CI: 1.56–4.60). In contrast, peer-led models showed no significant effect (OR = 1.09, 95% CI: 0.76–1.55) (Figure 7B). This finding is a primary driver of the extreme heterogeneity (*i*^2^ = 99.0%) observed in the main analysis, indicating that the choice of delivery model is a critical determinant of success for PrEP uptake.

For PrEP willingness, subgrouping by study design also showed a significant difference (*p* = 0.0002). Cross-sectional studies reported a substantially larger effect (OR = 5.90, 95% CI: 4.88–7.13) compared to cohort studies (OR = 2.39, 95% CI: 1.54–3.72) (Figure 8A). This suggests that while both designs show a positive impact, the magnitude of effect may be influenced by study methodology, contributing to the high overall heterogeneity. Finally, the sensitivity analysis for PrEP-related outcomes was highly revealing. The initial analysis, which was heavily skewed by a single large study (25), showed a negative effect (OR = 0.54) (Figure 8B). However, after excluding this review, the pooled effect reversed dramatically to become strongly positive and statistically significant (OR = 4.74, 95% CI: 2.30–9.76). Crucially, heterogeneity was eliminated, with *i*^2^ dropping from 92 to 0%. This confirms that community-based models are indeed effective for improving PrEP-related outcomes, and the contrary finding in the main analysis was an artifact of a single outlier study.

#### Assessment of publication bias

**Figure 9:**
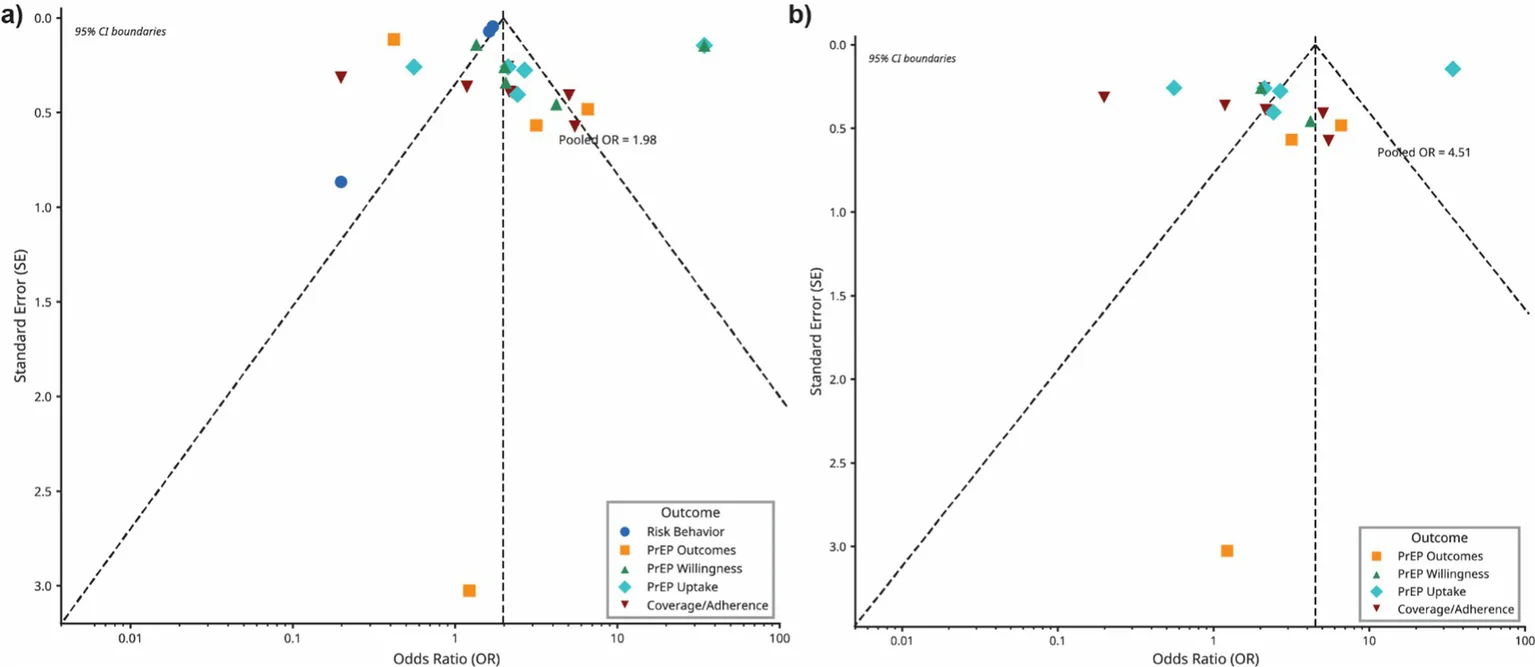
Funnel plots for the visual evaluation of publication bias. The figure displays the study effect size (odds ratio) against a measure of precision (standard error) for both main and subgroup analyses. **(A)** Main outcomes: This plot combines all studies across different outcome categories to visualize the overall distribution and potential for publication bias or heterogeneity. The observed asymmetry, particularly the gap of smaller studies in the lower-left quadrant, suggests a potential for publication bias. **(B)** Subgroups: This plot displays the same studies but differentiates them by outcome category (PrEP Outcomes, PrEP Uptake, PrEP Willingness, and Coverage/Adherence). It illustrates that much of the asymmetry seen in the main plot is attributable to between-study heterogeneity, as studies tend to cluster within their respective subgroups.

Visual assessment of the combined funnel plot for main outcomes and subgroups (Figure 9) indicates potential asymmetry, with a relative absence of smaller studies showing negative effects. This pattern, commonly observed in meta-analyses of behavioral interventions, suggests a possibility of publication bias where null or negative findings are less likely to be published. However, it is critical to note that our pre-specified subgroup analyses, visualized in Figure 9B, serve as a powerful internal check against this limitation. The extreme heterogeneity (*i*^2^ up to 99%) that drives the funnel plot asymmetry in the main analysis is successfully explained by key moderators when the data are stratified by outcome. As shown in Figure 9B, studies cluster together by outcome type, significantly reducing the impression of asymmetry within each domain. For instance, our analyses found that geographic region explained heterogeneity in coverage/adherence (subgroup difference *p* = 0.002), intervention type explained uptake heterogeneity (*p* < 0.00001), and study design explained willingness heterogeneity (*p* = 0.0002). Furthermore, a sensitivity analysis for PrEP-related outcomes showed that an apparent negative effect was entirely attributable to a single large study; upon its exclusion, heterogeneity vanished (*i*^2^ = 0%), and a strong positive effect emerged. Consequently, while publication bias cannot be entirely ruled out, our analytical approach provides a more nuanced and reliable interpretation than the pooled estimate alone. By identifying and explaining the sources of heterogeneity through subgroup analysis (Figure 9B), we demonstrate that the central finding of this review is not a single overall effect size but rather the demonstration of what works, where, and for whom, a conclusion that is less susceptible to distortion by publication bias.

## Discussion

This systematic review and meta-analysis provide the first comprehensive quantitative synthesis of the global evidence for community-based PrEP delivery models for MSM. Our findings reveal a nuanced picture: while community-based models are exceptionally effective at increasing willingness to use PrEP, their impact on the crucial implementation outcomes of uptake and adherence is highly variable. The novelty of this review is not in the pooled estimates, which are confounded by extreme heterogeneity, but in the use of subgroup analyses to dissect this variation and identify the specific contextual factors that drive success. This approach fills a critical gap in the literature, moving beyond the simple question of *if* community models work to the more important question of *what* works, and *for whom*. Previous works have been unable to provide this level of insight. For instance, the scoping review by Hillis et al. (15) mapped the landscape of PrEP delivery but explicitly did not provide a quantitative synthesis of effectiveness. Other meta-analyses have been geographically restricted, such as the work by Wang et al. (13) which focused only on the United States. Our study is the first to synthesize global data and, crucially, to treat the observed heterogeneity not as a limitation but as a central finding to be explained. By conducting pre-planned subgroup analyses, a standard method for investigating sources of variation in meta-analyses (45). We demonstrated that much of the inconsistency in outcomes could be systematically explained. Though our finding that the effectiveness of community-based models for adherence and coverage is significantly greater in high-income areas aligns with other research showing that location is a key moderator of PrEP outcomes (14).

PrEP willingness analysis suggests that community-based models are effective in improving attitudes and reducing barriers to PrEP acceptance. Willingness is a critical prerequisite for actual uptake, and these findings suggest that successfully decentralizing services addresses key psychological and logistical hurdles. Thus, community-based delivery is a strongly supported strategy for increasing PrEP willingness among MSM, forming a foundational step for successful prophylaxis programs. PrEP uptake analysis indicates that, on average, the current evidence does not conclusively demonstrate that community-based delivery models improve PrEP uptake compared to standard care. This pattern highlights that the success of community-based PrEP uptake is highly program-dependent. The lack of a statistically significant pooled effect is a direct consequence of these divergent results. The extreme heterogeneity suggests that factors such as program design, target population, integration with other services, and stigma mitigation efforts likely play a decisive role in determining success. The results underscore the critical need to identify and replicate the active components of highly successful programs like that of Di Ciaccio et al. (29), while investigating the barriers faced in contexts like that of Hojilla et al. (31).

PrEP coverage and adherence analysis suggest that, on average, community-based delivery models did not lead to a consistent, statistically meaningful improvement in these outcomes compared to standard care. This pattern underscores significant regional and programmatic differences in effectiveness. The lack of a significant overall pooled effect likely stems from methodological disparities among studies, including diverse definitions of adherence and coverage metrics, as well as differences in target populations and intervention components (e.g., peer-led, telehealth-integrated). The null finding at the pooled level contrasts with clinical expectations and highlights that “community-based delivery” is not a singular intervention. The observed diversity implies that these strategies require careful customization, considering cultural context, service integration, and support structures to effectively improve participation and compliance. The considerable heterogeneity further underscores the necessity for standardized metrics and detailed reporting of intervention components in future research to identify the active ingredients of successful programs.

On the other hand, three studies suggest that the “PrEP-related outcomes” measured (such as retention or adherence) may be highly sensitive to the specific clinical setting or population group. The dominance of a single large-scale study in the fixed-effects model suggests that while smaller community-based programs can be highly successful, scaling these models to larger populations, as seen in the Hendrickson study, may present significant challenges in maintaining efficacy compared to standard clinical care. Meanwhile, the change in risk behavior analysis challenges concerns regarding widespread “risk compensation” following PrEP initiation. Hence, the significant overall finding suggests that engagement with community-based PrEP services, which often integrate comprehensive sexual health education and counseling, is more commonly associated with sustained or improved risk reduction practices. The evidence indicates that community-based PrEP programs support HIV prevention efforts by promoting positive or neutral changes in sexual risk behavior (46, 47).

The most striking finding from our subgroup analysis is the powerful effect of telehealth interventions on PrEP uptake (OR = 25.20). This demonstrates that the specific “active ingredients” of a program are the true drivers of effectiveness. While peer-led models showed no significant effect on uptake, telehealth models, which often streamline access to prescriptions and reduce logistical barriers, were exceptionally successful. This aligns with a growing body of evidence supporting the effectiveness of telehealth for PrEP delivery (48). Similarly, our sensitivity analysis for PrEP-related outcomes revealed that an initially negative finding was an artifact of a single, large-scale study; its exclusion reversed the effect and eliminated heterogeneity, confirming the effectiveness of smaller, more focused community programs. This meta-analysis has several strengths, including its comprehensive global scope and rigorous methodology. However, limitations must be acknowledged, as the quality of the included evidence was mixed, and the observational nature of most studies precludes definitive causal inference. Furthermore, while our subgroup analyses explained a significant portion of the heterogeneity, residual variation remains, suggesting other unmeasured factors are at play.

Our findings support a differentiated, evidence-based approach to PrEP delivery rather than a one-size-fits-all community-based strategy. While telehealth models demonstrated exceptional effectiveness for PrEP uptake (OR = 25.20), community-based models remain a powerful tool for generating demand and improving willingness, as demonstrated by the consistently positive effects observed across all five willingness studies. Health systems and funders should therefore consider hybrid architectures that preserve community-based engagement and education while integrating streamlined telehealth or pharmacy-led pathways for prescription and clinical follow-up. This preserves the strengths of community connection while addressing the implementation bottlenecks observed for uptake and adherence. Furthermore, the effectiveness of community-based models should not be discounted for populations beyond MSM. Emerging evidence from Namibia demonstrates that community and hybrid community-clinic models can achieve high PrEP education coverage and moderate uptake among adolescent girls and young women, a population facing distinct structural barriers. This suggests that the contextual drivers of success, trust, accessibility, and reduced stigma, may translate across key populations, even if the optimal delivery modality differs. Consequently, the high heterogeneity observed in this review should be interpreted not as a failure of community-based approaches, but as evidence that their design must be carefully matched to local epidemiological, cultural, and health system contexts (49).

For outcomes like adherence, our findings suggest that program design must be tailored to the geographic and cultural context, as models effective in high-income countries may not be directly transferable to low- and middle-income countries without adaptation. To bridge the critical gap between high willingness and inconsistent uptake, programs should focus on implementing hybrid models that leverage the strengths of different approaches. For example, using community- and peer-led platforms for demand generation and education, while integrating streamlined telehealth or pharmacy-led pathways for prescription and clinical follow-up, may prove to be the most effective strategy. Future research should move away from monolithic evaluations and instead use implementation science frameworks to compare the effectiveness of these specific, differentiated models.

### Limitations

This review has several limitations that should be considered when interpreting the findings. First, the restriction to English-language publications may have excluded relevant evidence from non-Anglophone regions with high HIV burden, including Latin America, Francophone Africa, and parts of Asia, potentially introducing language bias and limiting the generalizability of our conclusions. Second, the majority of included studies were observational (cross-sectional or cohort), which limits causal inference and may leave residual confounding from unmeasured variables such as socioeconomic status, stigma, or healthcare access. Third, the search strategy primarily used “MSM” terminology, which may not have captured all studies using “GBMSM” or related variants, potentially introducing terminology bias in study retrieval. Fourth, we observed extreme statistical heterogeneity (I^2^ up to 99%) across most outcomes, indicating that study contexts, populations, and intervention designs varied widely; while subgroup analyses explained a portion of this variation, residual heterogeneity persisted. Furthermore, definitions of adherence, coverage, and retention were not standardized across studies, complicating direct comparison. Additionally, the review primarily captures evidence from oral TDF/FTC-based PrEP programs, as newer formulations (TAF/FTC, LA-CAB) were not widely represented in community-based implementation studies within the search timeframe. This heterogeneity in PrEP modality may contribute to the observed variation in outcomes. Finally, for PrEP-related outcomes, a single large-scale study (25) dominated the pooled estimate, and its exclusion reversed the direction of effect; this underscores the sensitivity of meta-analytic conclusions to individual influential studies and warrants caution in interpreting the main pooled estimate for this outcome.

## Conclusion

In conclusion, this meta-analysis provides a nuanced understanding of community-based PrEP delivery for MSM. We establish that these models are a powerful tool for engaging MSM and boosting willingness to use PrEP, while also demonstrating that their impact on uptake and adherence is highly variable and context-dependent. The high degree of heterogeneity across studies is not random noise but a clear signal that effectiveness is contingent on specific, context-sensitive design choices, most notably the integration of telehealth for uptake and tailoring of adherence support to local health system capacity. Rather than abandoning community-based approaches, our findings indicate that the most promising path forward lies in hybrid models that combine community-driven demand generation with streamlined clinical delivery pathways. Future policy, funding, and research should focus on disentangling the active ingredients of successful programs and adapting them to diverse socio-cultural and health system contexts, rather than applying generic community-based templates.
