# Supplementary material for: Effectiveness of community-based PrEP delivery for men who have sex with men: a global systematic review and meta-analysis
Source: Front Public Health. 2026 Sep 10;14:1868507. doi: 10.3389/fpubh.2026.1868507 (PMC13602209; doi:10.3389/fpubh.2026.1868507)
Supplement: Supplementary file 1 [file Table_10.docx]

**Supplementary Table S9.** PrEP-related outcomes

| **Study (First Author, Year)** | **log[Odds Ratio]** | **Standard Error (SE)** | **Weight (%)** | **Odds Ratio** | **95% Confidence Interval** |
| --- | --- | --- | --- | --- | --- |
| Erenrich et al., 2024 | 1.89 | 0.49 | 5.9% | 6.62 | [2.53, 17.29] |
| Gan et al., 2025 | 0.198 | 3.03 | 0.2% | 1.22 | [0.00, 462.52] |
| Hendrickson et al., 2025 | –0.872 | 0.126 | 89.5% | 0.42 | [0.33, 0.54] |
| Wilton et al., 2018 | 1.15 | 0.57 | 4.4% | 3.16 | [1.03, 9.65] |

***Note: Random-effects meta-analysis summary estimate. Overall Odds Ratio*** *= 0.54, 95% CI [0.43, 0.68], Z=5.19, P<0.00001.* ***Heterogeneity statistics****: τ2=2.73, χ2=39.95, df = 3, P<0.00001, I2=92%*
