# Supplementary material for: Effectiveness of community-based PrEP delivery for men who have sex with men: a global systematic review and meta-analysis
Source: Front Public Health. 2026 Sep 10;14:1868507. doi: 10.3389/fpubh.2026.1868507 (PMC13602209; doi:10.3389/fpubh.2026.1868507)
Supplement: Supplementary file 2 [file Table_11.docx]

**Supplementary S10**. Change in risk behavior

| **Study (First Author, Year)** | **log[Odds Ratio]** | **Standard Error (SE)** | **Weight (%)** | **Odds Ratio** | **95% Confidence Interval** |
| --- | --- | --- | --- | --- | --- |
| Chakrapani et al., 2021 | 0.53 | 0.0459 | 71.0% | 1.70 | [1.55, 1.86] |
| Phanuphak et al., 2018 | –1.615 | 0.866 | 0.2% | 0.20 | [0.04, 1.09] |
| Rana et al., 2018 | 0.476 | 0.072 | 28.8% | 1.61 | [1.40, 1.85] |

***Note:*** *Random-effects meta-analysis summary estimate. Overall Odds Ratio = 1.67, 95% CI [1.54, 1.8], Z=13.19, P<0.00001. Heterogeneity statistics: τ2=0.02, χ2=6.43, df = 2, P=0.04, I2=69%*
