# Supplementary material for: Effectiveness of community-based PrEP delivery for men who have sex with men: a global systematic review and meta-analysis
Source: Front Public Health. 2026 Sep 10;14:1868507. doi: 10.3389/fpubh.2026.1868507 (PMC13602209; doi:10.3389/fpubh.2026.1868507)
Supplement: Supplementary file 3 [file Table_12.docx]

**Supplementary Table S11**. Subgroup analysis

PrEP coverage and adherence by geographic region

| **Study (First Author, Year)** | **log[Odds Ratio]** | **Standard Error (SE)** | **Weight (%)** | **Odds Ratio** | **95% Confidence Interval** |
| --- | --- | --- | --- | --- | --- |
| ***Low and middle-income*** |  |  |  |  |  |
| Eubanks, 2022 | 0.7701 | 0.389 | 12.1% | 2.16 | [1.01, 4.63] |
| Laurent et al., 2021 | -1.6094 | 0.3162 | 18.3% | 0.20 | [0.11, 0.37] |
| Songtaweesin et al., 2020 | 0.166 | 0.364 | 13.8% | 1.18 | [0.58, 2.41] |
| Zhang et al., 2025 | 1.695 | 0.573 | 5.6% | 5.45 | [1.77, 16.74] |
| **Subtotal (95% CI)** |  |  | **49.9%** | **0.84** | **[0.58, 1.23]** |
| ***High income*** |  |  |  |  |  |
| Erenrich et al., 2024 | 1.611 | 0.41 | 10.9% | 5.01 | [2.24, 11.19] |
| Robles et al., 2022 | 0.751 | 0.259 | 27.3% | 2.12 | [1.28, 3.52] |
| Wheeler et al., 2019 | 0.797 | 0.392 | 11.9% | 2.22 | [1.03, 4.78] |
| **Subtotal (95% CI)** |  |  | **50.1%** | **2.58** | **[1.78, 3.76]** |
| **Total (95% CI)** |  |  | **100.0%** | **1.48** | **[1.13, 1.93]** |

***Note:*** *Random-effects meta-analysis summary estimate. Overall Odds Ratio = 1.48, 95% CI [1.13, 1.93], Z = 2.89, P < 0.004. Heterogeneity statistics: τ² = N/A, χ² = 58.39, df = 6, P < 0.00001, I² = 90%. Test for subgroup differences: χ² = 17.04, df = 1, P < 0.0001, I² = 94.1%*

Subgroup analysis of PrEP uptake by intervention type

| **Study (First Author, Year)** | **log[Odds Ratio]** | **Standard Error (SE)** | **Weight (%)** | **Odds Ratio** | **95% Confidence Interval** |
| --- | --- | --- | --- | --- | --- |
| ***Peer_led*** |  |  |  |  |  |
| Hojilla et al., 2018 | -0.5798 | 0.258 | 15.5% | 0.56 | [0.34, 0.93] |
| Robles et al., 2022 | 0.751 | 0.259 | 15.4% | 2.12 | [1.28, 3.52] |
| **Subtotal (95% CI)** |  |  | **30.9%** | **1.09** | **[0.76, 1.55]** |
| ***Telehealth*** |  |  |  |  |  |
| Di Ciaccio et al., 2024 | 3.53 | 0.145 | 49.2% | 34.12 | [25.68, 45.34] |
| Ding et al., 2016 | 0.875 | 0.404 | 6.3% | 2.40 | [1.09, 5.30] |
| **Subtotal (95% CI)** |  |  | **55.5%** | **25.20** | **[19.29, 32.93]** |
| ***Hybrid*** |  |  |  |  |  |
| Van Dijk., 2020 | 0.986 | 0.276 | 13.6% | 2.68 | [1.56, 4.60] |
| **Subtotal (95% CI)** |  |  | **13.6%** | **2.68** | **[1.56, 4.60]** |
| **Total (95% CI)** |  |  | **100.0%** | **7.03** | **[5.76, 8.58]** |

***Note:*** *Random-effects meta-analysis summary estimate. Overall Odds Ratio = 7.03, 95% CI [5.76, 8.58], Z = 19.18, P < 0.00001. Heterogeneity statistics: τ² = N/A, χ² = 255.59, df = 4, P < 0.00001, I² = 98%. Test for subgroup differences: χ² = 204.08, df = 2, P < 0.00001, I² = 99.0%*

Subgroup analysis of PrEP willingness by study design

| **Study (First Author, Year)** | **log[Odds Ratio]** | **Standard Error (SE)** | **Weight (%)** | **Odds Ratio** | **95% Confidence Interval** |
| --- | --- | --- | --- | --- | --- |
| ***Cohort*** |  |  |  |  |  |
| Van Dijk et al., 2020 | 0.693 | 0.258 | 11.9% | 2.00 | [1.21, 3.32] |
| Yu et al., 2021 | 1.4351 | 0.4559 | 3.8% | 4.20 | [1.72, 10.26] |
| **Subtotal (95% CI)** |  |  | **15.7%** | **2.39** | **[1.54, 3.72]** |
| ***Cross-sectional*** |  |  |  |  |  |
| Di Ciaccio et al, 2022 | 3.53 | 0.145 | 37.6% | 34.12 | [25.68, 45.34] |
| Lim et al., 2017 | 0.3075 | 0.1405 | 40.0% | 1.36 | [1.03, 1.79] |
| Pierre et al., 2018 | 0.708 | 0.341 | 6.8% | 2.03 | [1.04, 3.96] |
| **Subtotal (95% CI)** |  |  | **84.3%** | **5.90** | **[4.88, 7.13]** |
| **Total (95% CI)** |  |  | **100.0%** | **5.12** | **[4.30, 6.10]** |

***Note:*** *Random-effects meta-analysis summary estimate. Overall Odds Ratio = 5.12, 95% CI [4.30, 6.10], Z = 18.38, P < 0.00001. Heterogeneity statistics: τ² = N/A, χ² = 280.98, df = 4, P < 0.00001, I² = 99%. Test for subgroup differences: χ² = 13.60, df = 1, P = 0.0002, I² = 92.6%*

Sensitivity test of PrEP-related outcomes

| **Study (First Author, Year)** | **log[Odds Ratio]** | **Standard Error (SE)** | **Weight (%)** | **Odds Ratio** | **95% Confidence Interval** |
| --- | --- | --- | --- | --- | --- |
| Erenrich et al., 2024 | 1.89 | 0.49 | 56.7% | 6.62 | [2.53, 17.29] |
| Gan et al., 2025 | 0.198 | 3.03 | 1.5% | 1.22 | [0.00, 462.52] |
| **Hendrickson et al., 2025** | -0.872 | 0.126 | - | **Not estimable** | - |
| Wilton et al., 2018 | 1.15 | 0.57 | 41.9% | 3.16 | [1.03, 9.65] |
| **Total (95% CI)** |  |  | **100.0%** | **4.74** | **[2.30, 9.76]** |

***Note:*** *Random-effects meta-analysis summary estimate. Overall Odds Ratio = 4.74, 95% CI [2.30, 9.76], Z = 4.22, P < 0.0001. Heterogeneity statistics: τ² = N/A, χ² = 1.17, df = 2, P = 0.56, I² = 0%*
