# Supplementary material for: Effectiveness of community-based PrEP delivery for men who have sex with men: a global systematic review and meta-analysis
Source: Front Public Health. 2026 Sep 10;14:1868507. doi: 10.3389/fpubh.2026.1868507 (PMC13602209; doi:10.3389/fpubh.2026.1868507)
Supplement: Supplementary file 4 [file Table_2.docx]

**Supplementary Table S1.** Complete search syntax for database queries

| Database | Date of Search | Search Syntax | Filters Applied | Results |
| --- | --- | --- | --- | --- |
| PubMed | October 15, 2025 | ("Community-Based"[MeSH Terms] OR "community-based"[Title/Abstract]) AND ("Pre-Exposure Prophylaxis"[MeSH Terms] OR "PrEP"[Title/Abstract]) AND ("Men Who Have Sex with Men"[MeSH Terms] OR "MSM"[Title/Abstract]) NOT (review[Publication Type] OR survey[Title/Abstract]) | Publication date: 2015/01/01–2025/12/31; Language: English; Article type: Journal Article | 732 |
| Web of Science (Core Collection) | October 15, 2025 | TS=("community-based" OR "community based") AND TS=("pre-exposure prophylaxis" OR "PrEP") AND TS=("men who have sex with men" OR "MSM") NOT TS=("review" OR "survey") | Timespan: 2015–2025; Language: English; Document type: Article | 145 |
| Scopus | October 15, 2025 | TITLE-ABS-KEY("community-based" OR "community based") AND TITLE-ABS-KEY("pre-exposure prophylaxis" OR "PrEP") AND TITLE-ABS-KEY("men who have sex with men" OR "MSM") AND NOT TITLE-ABS-KEY("review" OR "survey") | Publication year: 2015–2025; Language: English; Article type: Journal Article | 112 |
| ScienceDirect | October 15, 2025 | ("community-based") AND ("pre-exposure prophylaxis" OR "PrEP") AND ("men who have sex with men" OR "MSM") | Year: 2015–2025; Article type: Research articles | 98 |
| Springerlink | October 15, 2025 | "community-based" AND ("pre-exposure prophylaxis" OR "PrEP") AND ("men who have sex with men" OR "MSM") | Date: 2015–2025; Language: English; Content type: Article | 52 |
| Embase | October 15, 2025 | ('community based'/exp OR 'community-based':ti,ab) AND ('pre-exposure prophylaxis'/exp OR 'PrEP':ti,ab) AND ('men who have sex with men'/exp OR 'MSM':ti,ab) NOT ('review'/it OR 'survey':ti,ab) | Publication year: 2015–2025; Language: English; Publication type: Article | 28 |
| Google Scholar | October 15, 2025 | "community-based" "pre-exposure prophylaxis" OR "PrEP" "men who have sex with men" OR "MSM" | Custom range: 2015–2025 | Supplementary search |
| Total Records Before Duplicate Removal | - | - | - | 1,167 |
| Total Records After Duplicate Removal | - | - | - | **623** |
| Records Screened | - | - | - | **544** |
| Full-text Articles Assessed for Eligibility | - | - | - | 52 |
| Studies Included in Meta-analysis | - | - | - | 21 |

**Notes:**

1 Search Strategy Logic: The search combined controlled vocabulary (MeSH terms for PubMed, Emtree for Embase) with free-text keywords to maximize sensitivity and specificity.
2 Platform-Specific Adjustments: Each database required syntax modifications according to their search interface (e.g., TS= for Web of Science, TITLE-ABS-KEY for Scopus, /exp for Embase).
3 Exclusion Filters: Review articles and surveys were systematically excluded using publication type filters and keyword exclusions.
4 Time Frame: January 2015 to December 2025 was selected to capture contemporary evidence on PrEP implementation among MSM.
5 Google Scholar: Used as a supplementary search engine to identify grey literature and ensure comprehensive coverage.
