# Supplementary material for: Effectiveness of community-based PrEP delivery for men who have sex with men: a global systematic review and meta-analysis
Source: Front Public Health. 2026 Sep 10;14:1868507. doi: 10.3389/fpubh.2026.1868507 (PMC13602209; doi:10.3389/fpubh.2026.1868507)
Supplement: Supplementary file 5 [file Table_3.docx]

**Supplementary S2.** PRISMA 2020 checklist

| **Section and Topic** | **Item #** | **Checklist item** | **Location where item is reported** |
| --- | --- | --- | --- |
| **TITLE** |  |  |  |
| Title | 1 | Identify the report as a systematic review. | Page 1, Title |
| **ABSTRACT** |  |  |  |
| Abstract | 2 | See the PRISMA 2020 for Abstracts checklist. | Page 1, Abstract |
| **INTRODUCTION** |  |  |  |
| Rationale | 3 | Describe the rationale for the review in the context of existing knowledge. | Pages 2--3, Introduction |
| Objectives | 4 | Provide an explicit statement of the objective(s) or question(s) the review addresses. | Page 1, Abstract; Pages 2--3, Introduction (including PrEP care cascade framework) |
| **METHODS** |  |  |  |
| Eligibility criteria | 5 | Specify the inclusion and exclusion criteria for the review and how studies were grouped for the syntheses. | Page 4, Inclusion and exclusion criteria |
| Information sources | 6 | Specify all databases, registers, websites, organizations, reference lists and other sources searched or consulted to identify studies. Specify the date when each source was last searched or consulted. | Page 3, Review protocol and search strategy; Supplementary Table S1 |
| Search strategy | 7 | Present the full search strategies for all databases, registers and websites, including any filters and limits used. | Pages 3--4, Review protocol and search strategy; Supplementary Table S1 |
| Selection process | 8 | Specify the methods used to decide whether a study met the inclusion criteria of the review, including how many reviewers screened each record and each report retrieved, whether they worked independently, and if applicable, details of automation tools used in the process. | Pages 4--5, Study selection and eligibility criteria |
| Data collection process | 9 | Specify the methods used to collect data from reports, including how many reviewers collected data from each report, whether they worked independently, any processes for obtaining or confirming data from study investigators, and if applicable, details of automation tools used in the process. | Pages 4--5, Study selection and eligibility criteria |
| Data items | 10a | List and define all outcomes for which data were sought. Specify whether all results that were compatible with each outcome domain in each study were sought (e.g. for all measures, time points, analyses), and if not, the methods used to decide which results to collect. | Page 1, Abstract; Page 6, Overview of included studies; Pages 7--15, Meta-analysis; Supplementary Table S5 |
| Data items | 10b | List and define all other variables for which data were sought (e.g. participant and intervention characteristics, funding sources). Describe any assumptions made about any missing or unclear information. | Page 6, Overview of included studies; Supplementary Table S5 |
| Study risk of bias assessment | 11 | Specify the methods used to assess risk of bias in the included studies, including details of the tool(s) used, how many reviewers assessed each study and whether they worked independently, and if applicable, details of automation tools used in the process. | Pages 4--5, Study selection and eligibility criteria; Supplementary File S4 |
| Effect measures | 12 | Specify for each outcome the effect measure(s) (e.g. risk ratio, mean difference) used in the synthesis or presentation of results. | Pages 6--7, Data Analysis and Synthesis; Pages 7--15, Meta-analysis subsections |
| Synthesis methods | 13a | Describe the processes used to decide which studies were eligible for each synthesis (e.g. tabulating the study intervention characteristics and comparing against the planned groups for each synthesis (item #5)). | Page 4, Inclusion and exclusion criteria; Page 6, Overview of included studies |
| Synthesis methods | 13b | Describe any methods required to prepare the data for presentation or synthesis, such as handling of missing summary statistics, or data conversions. | Pages 6--7, Data Analysis and Synthesis (random-effects models, subgroup analyses, sensitivity analyses) |
| Synthesis methods | 13c | Describe any methods used to tabulate or visually display results of individual studies and syntheses. | Pages 7--15, Meta-analysis (forest plots: Figures 2--6); Table 1; Supplementary Tables S6--S10 |
| Synthesis methods | 13d | Describe any methods used to synthesize results and provide a rationale for the choice(s). If meta-analysis was performed, describe the model(s), method(s) to identify the presence and extent of statistical heterogeneity, and software package(s) used. | Pages 6--7, Data Analysis and Synthesis (random-effects models, I², Q statistic, τ²) |
| Synthesis methods | 13e | Describe any methods used to explore possible causes of heterogeneity among study results (e.g. subgroup analysis, meta-regression). | Pages 6--7, Data Analysis and Synthesis; Pages 15--16, Subgroup and Sensitivity Analyses; Figure 7a--7d |
| Synthesis methods | 13f | Describe any sensitivity analyses conducted to assess robustness of the synthesized results. | Pages 6--7, Data Analysis and Synthesis; Pages 15--16, Subgroup and Sensitivity Analyses; Figure 7d |
| Reporting bias assessment | 14 | Describe any methods used to assess risk of bias due to missing results in a synthesis (arising from reporting biases). | Pages 16--17, Assessment of publication bias; Figures 8a--8b |
| Certainty assessment | 15 | Describe any methods used to assess certainty (or confidence) in the body of evidence for an outcome. | Not conducted; acknowledged as a limitation in the Limitations section (Page 19) |
| **RESULTS** |  |  |  |
| Study selection | 16a | Describe the results of the search and selection process, from the number of records identified in the search to the number of studies included in the review, ideally using a flow diagram. | Pages 4--5, Study selection and eligibility criteria; Figure 1 (Page 5) |
| Study selection | 16b | Cite studies that might appear to meet the inclusion criteria, but which were excluded, and explain why they were excluded. | Pages 4--5, Study selection and eligibility criteria |
| Study characteristics | 17 | Cite each included study and present its characteristics. | Page 6, Overview of included studies; Supplementary Table S5 |
| Risk of bias in studies | 18 | Present assessments of risk of bias for each included study. | Pages 4--5, Study selection and eligibility criteria; Supplementary File S4 |
| Results of individual studies | 19 | For all outcomes, present, for each study: (a) summary statistics for each group (where appropriate) and (b) an effect estimate and its precision (e.g. confidence/credible interval), ideally using structured tables or plots. | Pages 8--15, Figures 2--6; Supplementary Tables S6--S10 |
| Results of syntheses | 20a | For each synthesis, briefly summarize the characteristics and risk of bias among contributing studies. | Pages 7--15, Meta-analysis subsections |
| Results of syntheses | 20b | Present results of all statistical syntheses conducted. If meta-analysis was done, present for each the summary estimate and its precision (e.g. confidence/credible interval) and measures of statistical heterogeneity. If comparing groups, describe the direction of the effect. | Pages 7--15, Meta-analysis subsections; Figures 2--6; Table 1 |
| Results of syntheses | 20c | Present results of all investigations of possible causes of heterogeneity among study results. | Table 1 (Page 7); Pages 15--16, Subgroup and Sensitivity Analyses; Figures 7a--7d |
| Results of syntheses | 20d | Present results of all sensitivity analyses conducted to assess the robustness of the synthesized results. | Pages 15--16, Subgroup and Sensitivity Analyses; Figure 7d |
| Reporting biases | 21 | Present assessments of risk of bias due to missing results (arising from reporting biases) for each synthesis assessed. | Pages 16--17, Assessment of publication bias; Figures 8a--8b |
| Certainty of evidence | 22 | Present assessments of certainty (or confidence) in the body of evidence for each outcome assessed. | Not conducted; acknowledged as a limitation in the Limitations section (Page 19) |
| **DISCUSSION** |  |  |  |
| Discussion | 23a | Provide a general interpretation of the results in the context of other evidence. | Pages 17--19, Discussion |
| Discussion | 23b | Discuss any limitations of the evidence included in the review. | Page 19, Limitations section; Pages 17--19, Discussion |
| Discussion | 23c | Discuss any limitations of the review processes used. | Page 19, Limitations section |
| Discussion | 23d | Discuss implications of the results for practice, policy, and future research. | Pages 18--19, Discussion; Page 19, Limitations section; Page 20, Conclusion |
| **OTHER INFORMATION** |  |  |  |
| Registration and protocol | 24a | Provide registration information for the review, including register name and registration number, or state that the review was not registered. | Not registered; stated in Methods (Page 3, Review protocol and search strategy) |
| Registration and protocol | 24b | Indicate where the review protocol can be accessed, or state that a protocol was not prepared. | Protocol not prepared; stated in Methods (Page 3, Review protocol and search strategy) |
| Registration and protocol | 24c | Describe and explain any amendments to information provided at registration or in the protocol. | Not applicable (no protocol prepared) |
| Support | 25 | Describe sources of financial or non-financial support for the review, and the role of the funders or sponsors in the review. | Page 20, Funding |
| Competing interests | 26 | Declare any competing interests of review authors. | Page 20, Conflict of interests |
| Availability of data, code and other materials | 27 | Report which of the following are publicly available and where they can be found: template data collection forms; data extracted from included studies; data used for all analyses; analytic code; any other materials used in the review. | Page 20, Consent for publication and availability of data and material |

***Note:*** *This checklist has been updated to reflect the revised manuscript structure, including the reordered Results section (PrEP care cascade sequence), the new Limitations section, and corrected page number references. Source: Page MJ, McKenzie JE, Bossuyt PM, Boutron I, Hoffmann TC, Mulrow CD, et al. The PRISMA 2020 statement: an updated guideline for reporting systematic reviews. BMJ 2021;372:n71. doi: 10.1136/bmj.n71.*
