# Supplementary material for: Effectiveness of community-based PrEP delivery for men who have sex with men: a global systematic review and meta-analysis
Source: Front Public Health. 2026 Sep 10;14:1868507. doi: 10.3389/fpubh.2026.1868507 (PMC13602209; doi:10.3389/fpubh.2026.1868507)
Supplement: Supplementary file 6 [file Table_4.docx]

**Supplementary S3.** JBI critical appraisal checklist for systematic reviews and research synthesis

| Reviewers: | WX, IA, AT | Date: | Nov 18, 2025 |
| --- | --- | --- | --- |
| Author: | Teeti et al. | Year: | 2025 |

| **Criteria** | **Yes** | **No** | **Unclear** | **Not applicable** |
| --- | --- | --- | --- | --- |
| 1. Is the review question clearly and explicitly stated? | **X** |  |  |  |
| 2. Were the inclusion criteria appropriate for the review question? | **X** |  |  |  |
| 3. Was the search strategy appropriate? | **X** |  |  |  |
| 4. Were the sources and resources used to search for studies adequate? | **X** |  |  |  |
| 5. Were the criteria for appraising studies appropriate? | **X** |  |  |  |
| 6. Was critical appraisal conducted by two or more reviewers independently? | **X** |  |  |  |
| 7. Were there methods to minimize errors in data extraction? | **X** |  |  |  |
| 8. Were the methods used to combine studies appropriate? | **X** |  |  |  |
| 9. Was the likelihood of publication bias assessed? | **X** |  |  |  |
| 10. Were recommendations for policy and/or practice supported by the reported data? | **X** |  |  |  |
| 11. Were the specific directives for new research appropriate? | **X** |  |  |  |

**Overall appraisal:** √ Include	☐ Exclude	☐ Seek further info

***Comments (Including reason for exclusion):*** *This systematic review and meta-analysis meets all JBI critical appraisal criteria. The review question is clearly stated in the abstract and introduction, focusing on the effectiveness of community-based PrEP delivery models for MSM across five key outcome domains. The inclusion and exclusion criteria are explicitly defined and appropriate for the research question. A comprehensive search strategy was employed across six databases (PubMed, Web of Science, Scopus, ScienceDirect, Embase, and Google Scholar) with clearly described search terms. Study selection was conducted by two independent reviewers (WX and IA) with discrepancies resolved by a third reviewer (AT). Risk of bias was assessed using design-specific tools (Cochrane RoB 2 for RCTs and NIH Quality Assessment Tool for observational studies). Random-effects models were appropriately used given the substantial heterogeneity observed. Publication bias was assessed via funnel plot analysis. The recommendations for policy and practice are directly supported by the meta-analytic findings, and specific directions for future research are provided.*
