# Supplementary material for: Effectiveness of community-based PrEP delivery for men who have sex with men: a global systematic review and meta-analysis
Source: Front Public Health. 2026 Sep 10;14:1868507. doi: 10.3389/fpubh.2026.1868507 (PMC13602209; doi:10.3389/fpubh.2026.1868507)
Supplement: Supplementary file 7 [file Table_5.docx]

**Supplementary S4**. Risk of bias tools

**A. Randomized Controlled Trials (RCTs)**

Tool: Cochrane RoB 2.0

| **Study ID** | **Randomization** | **Deviations** | **Missing Data** | **Measurement** | **Overall Risk** | **Rationale / Critical Appraisal** |
| --- | --- | --- | --- | --- | --- | --- |
| **Songtaweesin et al., 2020** | 🟢 Low | 🟡 Concerns | 🟢 Low | 🟡 Concerns | 🟡 **Concerns** | Open-label design (participants knew they were using the app); outcomes were self-reported. |
| **Ehrenrich et al., 2024** | 🟢 Low | 🟡 Concerns | 🟡 Concerns | 🟢 Low | 🟡 **Concerns** | Open-label telehealth intervention. Some differential attrition noted between arms. |

**B. Cohort Studies**

Tool: NIH quality assessment tool for observational cohort studies

| **Study ID** | **Selection** | **Confounding** | **Outcome** | **Attrition** | **Overall Risk** | **Rationale / Critical Appraisal** |
| --- | --- | --- | --- | --- | --- | --- |
| **Hojilla et al., 2018** | 🟢 Low | 🟢 Low | 🟢 Low | 🟢 Low | 🟢 **Low** | Used Electronic Medical Records (EMR) for objective retention/uptake data; minimal bias. |
| **Wheeler et al., 2019** | 🟢 Low | 🟢 Low | 🟢 Low | 🟢 Low | 🟢 **Low** | HPTN 073 protocol is rigorous; biological verification (DBS) used for adherence. |
| **Zhang et al., 2025** | 🟢 Low | 🟡 Moderate | 🟢 Low | 🟡 Moderate | 🟡 **Moderate** | Longitudinal design allows causal inference, but some residual confounding in transition data. |
| **Eubanks et al., 2022** | 🟢 Low | 🟢 Low | 🟢 Low | 🔴 High | 🔴 **High** | **Critical Flaw:** Reported high attrition (~57%), introducing significant attrition bias. |
| **Laurent et al., 2021** | 🟢 Low | 🟢 Low | 🟢 Low | 🟡 Moderate | 🟢 **Low** | Large multi-country sample (CohMSM); robust clinical data offsets moderate attrition. |
| **Phanuphak et al., 2018** | 🟢 Low | 🟡 Moderate | 🟡 Moderate | 🟢 Low | 🟡 **Moderate** | "Princess PrEP" model; risk behavior outcomes are self-reported (social desirability bias). |
| **Hendrickson et al., 2025** | 🟢 Low | 🟡 Moderate | 🟢 Low | 🟢 Low | 🟢 **Low** | Uses routine health data for modeling; high generalizability to the target population. |
| **Chakrapani et al., 2021** | 🟡 Moderate | 🟡 Moderate | 🟡 Moderate | 🟡 Moderate | 🟡 **Moderate** | Recruited from cruising sites (selection bias); self-reported sexual behavior. |

**C. Cross-Sectional Studies**

Tool: NIH quality assessment tool for cross-sectional studies

| **Study ID** | **Selection** | **Confounding** | **Outcome** | **Overall Risk** | **Rationale / Critical Appraisal** |
| --- | --- | --- | --- | --- | --- |
| **Rana et al., 2018** | 🟡 Moderate | 🟡 Moderate | 🟡 Moderate | 🟡 **Moderate** | Venue-based sampling (Gay Village); self-reported risk behavior. |
| **Wilton et al., 2018** | 🟢 Low | 🟢 Low | 🟢 Low | 🟢 **Low** | Clinic-based screening provides a defined denominator and clinical data. |
| **Gan et al., 2025** | 🔴 High | 🔴 High | 🟡 Moderate | 🔴 **High** | **Critical Flaw:** Extremely small sample size (N=18) makes results unreliable. |
| **Robles et al., 2022** | 🟡 Moderate | 🟡 Moderate | 🟡 Moderate | 🟡 **Moderate** | Online recruitment; self-reported PrEP uptake. |
| **van Dijk et al., 2020** | 🔴 High | 🟡 Moderate | 🔴 High | 🔴 **High** | Online convenience sample; likely over-represents motivated users; self-reported data. |
| **Ding et al., 2016** | 🟡 Moderate | 🟡 Moderate | 🟡 Moderate | 🟡 **Moderate** | Standard survey limitations; cross-sectional nature prevents causal inference. |
| **Di Ciaccio et al., 2024** | 🔴 High | 🟡 Moderate | 🔴 High | 🔴 **High** | Online survey; high selection bias; self-reported outcomes. |
| **Pierre et al., 2018** | 🔴 High | 🟡 Moderate | 🔴 High | 🔴 **High** | Discrete Choice Experiment (DCE) measures *hypothetical* willingness, not actual use. |
| **Yu et al., 2021** | 🔴 High | 🟡 Moderate | 🔴 High | 🔴 **High** | Large national online survey; strictly self-reported data without clinical verification. |
| **Di Ciaccio et al., 2022** | 🔴 High | 🟡 Moderate | 🔴 High | 🔴 **High** | (Same dataset as 2024); limitations of online recruitment apply. |
| **Lim et al., 2017** | 🔴 High | 🟡 Moderate | 🔴 High | 🔴 **High** | App-based recruitment (Grindr/Jack'd) excludes non-users; high selection bias. |

***Note:*** *Online surveys are rated "High Risk" for selection bias due to non-representative sampling.*
