# Supplementary material for: Effectiveness of community-based PrEP delivery for men who have sex with men: a global systematic review and meta-analysis
Source: Front Public Health. 2026 Sep 10;14:1868507. doi: 10.3389/fpubh.2026.1868507 (PMC13602209; doi:10.3389/fpubh.2026.1868507)
Supplement: Supplementary file 8 [file Table_7.docx]

**Supplementary S6.** PrEP willingness

| **Study (First Author, Year)** | **log[Odds Ratio]** | **Standard Error (SE)** | **Weight (%)** | **Odds Ratio** | **95% Confidence Interval** |
| --- | --- | --- | --- | --- | --- |
| Di Ciaccio et al., 2022 | 3.53 | 0.145 | 37.6% | 34.12 | [25.68, 45.34] |
| Lim et al., 2017 | 0.3075 | 0.1405 | 40.0% | 1.36 | [1.03, 1.79] |
| Pierre et al., 2018 | 0.708 | 0.341 | 6.8% | 2.03 | [1.04, 3.96] |
| Van Dijk et al., 2020 | 0.693 | 0.258 | 11.9% | 2.00 | [1.21, 3.32] |
| Yu et al., 2021 | 1.4351 | 0.4559 | 3.8% | 4.20 | [1.72, 10.26] |

***Note:*** *Fixed-effects meta-analysis summary estimate. Overall Odds Ratio = 5.12, 95% CI [4.30, 6.10], Z=18.38, P<0.00001. Heterogeneity statistics: χ2=280.98, df = 4, P<0.00001, I2=99%*
