# Supplementary material for: Effectiveness of community-based PrEP delivery for men who have sex with men: a global systematic review and meta-analysis
Source: Front Public Health. 2026 Sep 10;14:1868507. doi: 10.3389/fpubh.2026.1868507 (PMC13602209; doi:10.3389/fpubh.2026.1868507)
Supplement: Supplementary file 9 [file Table_8.docx]

**Supplementary Table S7.** PrEP uptake

| **Study (First Author, Year)** | **log[Odds Ratio]** | **Standard Error (SE)** | **Weight (%)** | **Odds Ratio** | **95% Confidence Interval** |
| --- | --- | --- | --- | --- | --- |
| Di Ciaccio et al., 2024 | 3.53 | 0.145 | 49.2% | 34.12 | [25.68, 45.34] |
| Ding et al., 2016 | 0.875 | 0.404 | 6.3% | 2.40 | [1.09, 5.30] |
| Hojilla et al., 2018 | -0.5798 | 0.258 | 15.5% | 0.56 | [0.34, 0.93] |
| Robles et al., 2022 | 0.751 | 0.259 | 15.4% | 2.12 | [1.28, 3.52] |
| Van Dijk et al., 2020 | 0.986 | 0.276 | 13.6% | 2.68 | [1.56, 4.60] |

***Note:*** *Random-effects meta-analysis summary estimate. Overall Odds Ratio = 3.06, 95% CI [0.55, 17.13], Z=1.27, P=0.20. Heterogeneity statistics: τ2=3.78, χ2=255.59, df = 4, P<0.00001, I2=98%*
