# Supplementary material for: Effectiveness of community-based PrEP delivery for men who have sex with men: a global systematic review and meta-analysis
Source: Front Public Health. 2026 Sep 10;14:1868507. doi: 10.3389/fpubh.2026.1868507 (PMC13602209; doi:10.3389/fpubh.2026.1868507)
Supplement: Supplementary file 10 [file Table_9.docx]

**Supplementary Table S8.** PrEP coverage and adherence

| **Study (First Author, Year)** | **log[Odds Ratio]** | **Standard Error (SE)** | **Weight (%)** | **Odds Ratio** | **95% Confidence Interval** |
| --- | --- | --- | --- | --- | --- |
| Ehrenrich et al., 2024 | 1.611 | 0.41 | 10.9% | 5.01 | [2.24, 11.19] |
| Eubanks et al., 2022 | 0.7701 | 0.389 | 12.1% | 2.16 | [1.01, 4.63] |
| Laurent et al., 2021 | -1.6094 | 0.3162 | 18.3% | 0.20 | [0.11, 0.37] |
| Robles et al., 2022 | 0.751 | 0.259 | 27.3% | 2.12 | [1.28, 3.52] |
| Songtaweesin et al., 2020 | 0.166 | 0.364 | 13.8% | 1.18 | [0.58, 2.41] |
| Wheeler et al., 2019 | 0.797 | 0.392 | 11.9% | 2.22 | [1.03, 4.78] |
| Zhang et al., 2025 | 1.695 | 0.573 | 5.6% | 5.45 | [1.77, 16.74] |

***Note: Random-effects meta-analysis summary estimate. Overall Odds Ratio*** *= 1.48, 95% CI [1.13, 1.93], Z=2.89, P=0.004.* ***Heterogeneity statistics****: τ2=1.16, χ2=58.39, df = 6, P<0.00001, I2=90%*
